# Comment on “Clinical Feasibility and Safety of Nasal Allergen Provocation Testing in Pediatric Allergic Rhinitis”

**DOI:** 10.1002/iid3.70512

**Published:** 2026-10-06

**Authors:** Abhinit Kumar, Ranjana Roy, Parappa Sajjan, Alok Bhatt, Karen Jaison

**Affiliations:** ^1^ Department of Psychiatry School of Medical Sciences and Research, Sharda University Greater Noida India; ^2^ Dr. D. Y. Patil Medical College, Hospital and Research Centre Dr. D. Y. Patil Vidyapeeth (Deemed‐to‐be‐University), Pimpri Pune Maharashtra India; ^3^ Department of Public Health, Malla Reddy Institute of Dental Sceinces Malla Reddy Vishwavidyapeeth (Deemed to be University) Hyderabad Telangana India; ^4^ Faculty of Pharmaceutical Sciences Graphic Era Hill University Dehradun India; ^5^ Centre for Promotion of Research Graphic Era Deemed University Dehradun India; ^6^ Saveetha Medical College and Hospital, Saveetha Institute of Medical and Technical Sciences Saveetha University Chennai India

Dear Editor,

Wang et al. report a prospective, single‐center evaluation of nasal allergen provocation testing (NAPT) in children with house‐dust‐mite allergic rhinitis [1]. Their large pediatric cohort provides useful procedural data, but we believe the findings support feasibility in a selected clinical population rather than definitive evidence of reproducibility, diagnostic performance, or safety across pediatric practice.

First, reproducibility was an explicit study objective, yet each participant appears to have undergone only one NAPT sequence. Completion of a single protocol and comparison with adult literature cannot estimate test‐retest agreement. The adult study cited by the authors evaluated repeated challenges and reported reproducibility, positive predictive value, and negative predictive value across consecutive tests [2]. A pediatric validation study should repeat NAPT after a prespecified interval, with stable disease and medication washout, and report agreement for positive status and eliciting‐dose category using kappa statistics or weighted agreement, together with within‐child changes in total nasal symptoms score (TNSS) and nasal flow.

Second, the absence of recorded adverse events among 290 tested children is reassuring but does not establish zero risk. With no events, the conventional rule‐of‐three gives an approximate upper 95% confidence boundary of 3/290, or 1.0% [3]. Generalizability is further limited by excluding children with previous severe reactions, uncontrolled asthma, major comorbidity, severe obstruction, or recent infection [1]. Safety reporting would be strengthened by prespecified definitions, severity grading, rescue treatment, immediate observation duration, delayed follow‐up, and confidence intervals. These details are particularly important when recommending wider clinical adoption.

Third, diagnostic and feasibility outcomes require clearer separation. Four of 294 recruits reacted to the negative control and did not undergo allergen challenge; consequently, the abstract reports 245/294 positive results (83.3%), whereas the Results section reports 245/290 (84.5%). Both denominators may be informative, but the target analysis population should be explicit. Moreover, NAPT positivity was defined partly by TNSS, and 92.2% of positive classifications were based on TNSS alone. Receiver‐operating‐characteristic analyses of individual symptoms against this composite classification, therefore, incorporate components of the outcome into the reference classification and may yield optimistic discrimination [4]. Similarly, the pilot probit table appears to present predicted response probabilities at allergen doses, not positive predictive values, which require an independent reference standard. The EAACI framework emphasizes standardized, combined subjective and objective assessment [5].

We suggest presenting this study as a valuable protocol‐feasibility and dose‐response investigation. Independent pediatric cohorts, repeated testing, blinded outcome assessment, externally defined clinical reference criteria, and structured safety surveillance are needed before the proposed concentrations and conclusions can be considered broadly validated.

## Author Contributions

**Abhinit Kumar:** conceptualization, funding acquisition, writing – original draft, investigation. **Ranjana Roy:** methodology, software, data curation. **Parappa Sajjan:** validation, formal analysis, supervision. **Alok Bhatt:** visualization, project administration. **Karen Jaison:** resources, writing – review and editing.

## Data Availability

The data that support the findings of this study are available on request from the corresponding author. The data are not publicly available due to privacy or ethical restrictions.
